# The League of Mercy

**Published:** 1904-12-24

**Authors:** 


### THE LEAGUE OF MERCY.

The fifth annual meeting of presidents of the League of Mercy was held on the 16th inst. at the Middlesex Hospital. At the opening of the meeting Mr. E. A. Hambro presided, vacating the chair later on in favour of his successor, Lord Mansfield, when the office bearers were elected for the ensuing year. In the course of the proceedings Sir William Collins read the following letter from Sir Arthur Bigge on behalf of the Prince of Wales, dated Marlborough House, December 15th:—

"Dear Sir William Collins,—As the presidents of the League of Mercy meet to-morrow, the Prince of Wales has directed me to ask you to be good enough to convey to the meeting hearty congratulations from the Princess of Wales, as Lady Grand President, and from himself, as Grand President of the League, upon the successful results achieved during the past year.

"His Royal Highness understands that probably the League will be able to hand over to the King's Hospital Fund upwards of £14,000.

"It is most gratifying to his Royal Highness, the President, to find not only that the amount is larger than last year's contribution, which was the largest on record, but also that the rate of progress has so greatly increased.

"His Royal Highness fully realises that these very satisfactory results are the outcome of earnest untiring efforts on the part of the workers of the League, to all of whom he offers his best thanks.

"His Royal Highness was glad to learn from those whom he had the pleasure of meeting at Marlborough House this summer how the League of Mercy was becoming more firmly established, its influence spreading widely, its work every day being better understood and appreciated.—Believe me, yours very truly, Arthur Bigge."

Sir William Collins having read this letter, announced the appointments made during the year, and reviewed the work that had been done. Experience, he said, had justified the original prediction that the League would be a success. The League of Mercy was reaching the very poorest, and securing the hearty co-operation of those who stood most in need of the London Hospitals. The success of the League was due to the personal interest taken in it by the Prince and Princess of Wales, to the numerous courtesies they had extended to the workers of the League, and to the indomitable perseverance of the presidents of the various districts.

Sir Henry Burdett, in making his annual financial statement, said that when the League was started it was thought to be nothing more than new machinery for collecting money in shillings for the hospitals. But it had proved itself something better than that. It had led to the devotion of time and personal interest in the cause of the sick. This year, for the first time, one of the districts had obtained more money than the maximum originally anticipated from each district. South Kensington had collected £1,222, an increase of £276 on last year's work; Fulham had raised £1,062. One of the Home counties—Berkshire—of which H.R.H. Princess Victoria was President, had raised £777 in 1904, against £16 in 1903. South Paddington had increased its contribution to £706, a success largely due to Miss Wakefield. The Strand Division had increased its contribution by as much as £319. The League would this year contribute over one-sixth of the total amount given to the London Hospitals by the King's Fund. The increase of small subscriptions testified to increasing public confidence. The total amount collected this year by the League of Mercy was £15,750, as compared with £13,138 last year, and this would enable them to increase their contribution to the King's Fund from £10,000 to £14,000.

Mrs. Moreing, hon. secretary to Princess Alexander of Teck, lady president of the Epsom-Esher district, described the work done in her district and its organisation by the Princess. The contributions from the district included 25s. collected in pennies by a charwoman, and £2 voluntarily collected by a little child in sixpences.

The Duchess of Somerset and Mr. Carl Hentschel also addressed the meeting.

We propose to publish a full list of the results attained by each district of the League in our next issue.

